# Bringing Multisectoral and Multidisciplinary Stakeholders Together to Optimize Environmental Health Research

**DOI:** 10.1029/2022GH000746

**Published:** 2023-02-20

**Authors:** A. S. Rosofsky, D. J. Vorhees

**Affiliations:** ^1^ Health Effects Institute Energy Boston MA USA

**Keywords:** research planning, multidisciplinary, oil and gas, community‐engaged

## Abstract

Incorporating multisectoral and multidisciplinary stakeholder perspectives into environmental health research planning and implementation can help increase the utility of the research. It can also enhance the likelihood of stakeholders using the research to inform decisions. Health Effects Institute (HEI) Energy, a funding and research organization, undertook a multiyear research planning process to build its stakeholder network and integrate their perspectives, knowledge, and priorities into research on potential exposures and health effects associated with unconventional oil and gas development. This commentary describes that process, lessons learned, and how stakeholder involvement shaped HEI Energy's inaugural program of research and associated stakeholder engagement.

## Key Points

- Stakeholder involvement in research planning and implementation can increase the utility and acceptance of the research
- We describe a process for research funders to partner with multisectoral stakeholders to plan and implement environmental health research

## 1 Introduction

Engaging stakeholders in environmental health research planning can help close the gap between research that interests only a small group of scientists and research that is widely useful for decision‐making (Boaz et al., 2018). Much of the literature on stakeholder participation in environmental health research has focused on community engagement in some or all aspects of the research process, as well as tools for researchers and communities to make research partnerships successful (Baldwin et al., 2021; English et al., 2018; Israel et al., 1998). Here, from the perspective of a funding organization, we describe a process for engaging multisectoral and multidisciplinary stakeholders in prioritizing and implementing environmental health research about unconventional oil and gas development (UOGD). The process falls within the GeoHealth paradigm by seeking to “blend earth, environmental, and health sciences while simultaneously informing policy and community action” (Hayhow et al., 2021).

Discourse around the risks and benefits of UOGD on community social, economic and environmental well‐being has been polarized (Elser et al., 2020; Graham et al., 2015; Ladd, 2013; Thomas et al., 2017). In this context, involving a broad range of stakeholders in research may promote its credibility to communities, industry, and policymakers with an interest in oil and gas operations, and its utility and suitability for addressing the needs of communities living near this development. Therefore, Health Effects Institute (HEI) Energy and its Research Committee (“the Committee”) developed and implemented a multiyear process to (a) identify stakeholders from multiple societal sectors, (b) engage them in identifying important knowledge gaps about community UOGD exposures and health effects and the research priorities that flow from these gaps, and (c) develop principles for successful stakeholder engagement from research planning to reporting.

**Figure 1:**
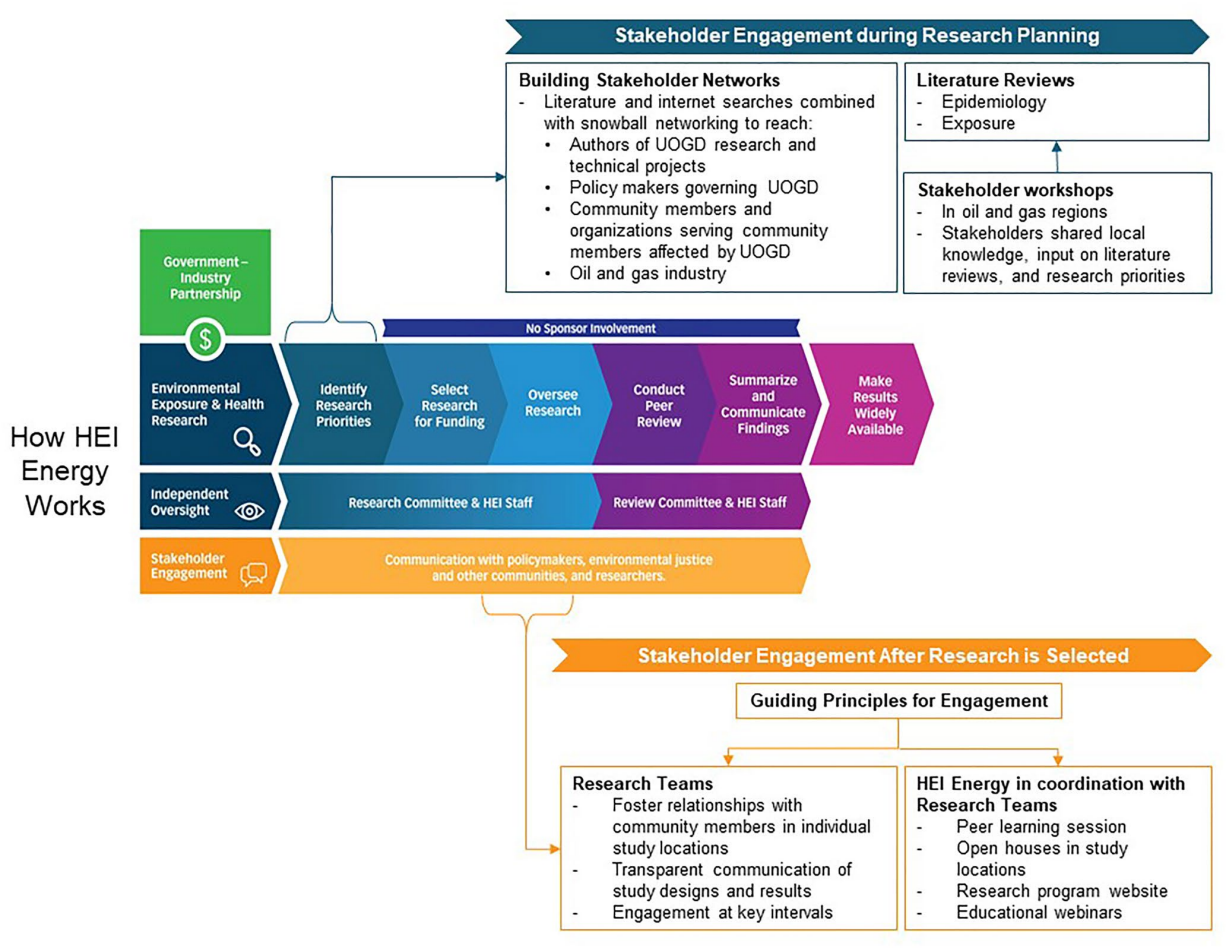
Stakeholder engagement process for research planning and implementation in the context of Health Effects Institute Energy's broader model for providing impartial, policy‐relevant science.

We summarize HEI Energy's application of this process within its broader model for providing impartial, policy‐relevant science (Figure 1) and offer lessons learned for funding organizations and research programs that want to knit stakeholder perspectives into research planning and implementation.

## 2 Stakeholder Workshops for Research Planning

In a series of six research planning workshops, HEI Energy convened stakeholders representing various sectors (academia, consulting companies and law firms, foundations, government agencies [federal, state, and local], nongovernmental organizations [including those connected to communities affected by UOGD], and the oil and natural gas industry) to ensure that HEI Energy funded research that is credible and widely useful for their decision‐making. The workshops provided an opportunity for stakeholders to come together and share their perspectives on the UOGD exposure and health literature, important knowledge gaps, corresponding research priorities about potential community exposures and health effects associated with UOGD, and criteria that HEI Energy should use to prioritize and fund research.

### 2.1 Participants

HEI Energy's stakeholder mapping involved several methods to identify stakeholder groups that would benefit from and contribute to the research program, as well as to define methods of engagement. Stakeholder groups can have both overlapping and competing priorities; therefore, HEI Energy encouraged balanced stakeholder group participation by directly contacting members of each stakeholder group and, for some community‐based non‐governmental organizations (NGOs) that could not otherwise be represented, covered expenses to allow for their participation in workshops (see Table S1 in Supporting Information S1 for list of workshop participants and Table S2 in Supporting Information S1 for participant distribution among stakeholder groups).

### 2.2 Format

Each workshop included presentations on topics relevant to HEI Energy's scope of research, coupled with facilitated open discussion and breakout group exercises centered around a set of charge questions (Table S3 in Supporting Information S1). Two of the workshops included poster sessions where stakeholders could discuss ongoing and past research directly with the investigators.

The presentations provided technical context for each workshop, while the group exercises fostered an environment where individuals from stakeholder groups that do not typically interact (e.g., industry representatives and community‐based NGOs) could come together and discuss their concerns and research. HEI Energy developed breakout group assignments ahead of each workshop to ensure a representative cross‐section of stakeholder groups. Committee members and HEI Energy staff were assigned to each group to facilitate discussion.

A single representative from each group, selected by the group, reported on highlights from their group's discussion to all workshop participants. The report‐back process elucidated shared priorities and highlighted where priorities diverged. The small group format allowed individuals who may feel uncomfortable speaking in large groups a more intimate setting for sharing their ideas. Participants (and anyone unable to attend the workshop) were also encouraged to share their perspectives on the charge questions in writing, providing a mechanism to share recommendations anonymously.

### 2.3 Major Insights

Stakeholders provided important insights that shaped the Committee's approach to assessing UOGD scientific literature and to selecting HEI Energy's inaugural program of research. The major insights identified in the workshops and literature reviews are found in Table 1.

**Table 1:** Criteria for Exposure Study Design and Implementation Identified by Health Effects Institute Energy Research Committee (in Alphabetical Order)

Criterion	Description
Brings value to and informs decision‐making	Is useful to communities in study areas, government officials, industry, and other stakeholders. Ideal study designs will be informed by successful engagement with the communities in study areas and other stakeholders
Broadly generalizable	Designed to be broadly generalizable across geographic regions, UOGD operating conditions, or communities over time, including periods of low and high UOGD activity, without sacrificing validity
Determines whether an exposure pathway links a UOGD process with a community	Links one or more chemical or non‐chemical agents directly released to the environment from a UOGD process to a potentially exposed community. The research allows for the detection of possible causal links between one or more UOGD processes (e.g., specific equipment, activity, or phase of development) and resulting human exposures. The study is designed to distinguish between agents released from UOGD and non‐UOGD sources
Provides understanding of temporal and spatial variability of exposure	Selected study locations and designs will substantially fill important gaps in understanding of variability in exposure conditions over temporal and spatial scales relevant for decision‐making by communities, regulators, industry, and other stakeholders
Optimizes use of the research budget by maximizing efficiency	Ensures that the research budget is spent on gathering data and information that is not already available (e.g., by incorporating or complementing existing data and information) and that prioritization and sequencing of data collection maintains a focus on exposures of possible concern
Useful for assessing health risk	Collects data or analyzes existing data (or establishes practical exposure assessment methodologies) that is useful for assessing the potential for human health effects at resolutions relevant for application in an epidemiology study or risk assessment

#### 2.3.1 Reviewing the Literature

The Committee reviewed the literature to assess the state‐of‐the science about potential community exposures and health effects associated with UOGD (HEI‐Energy Research Committee, 2019a, 2019b). The workshops shaped the objectives of the Committee's reviews of the epidemiology and exposure literature, ensuring that they were relevant and useful to various sectors. They also shaped the criteria that the Committee used to evaluate study quality and utility for assessing potential links between UOGD and community exposure and health. For example, workshop participants noted that much of the literature was not designed to identify complete exposure pathway(s), should one exist, between UOGD process(es) and a community(ies); the literature lacked statistical source apportionment, tracer release experiments, or other methods for understanding any connections between specific UOGD processes and community exposures. They also noted the lack of epidemiology studies quantifying exposure to specific chemicals or other possible health stressors. The Committee included these points as evaluative criteria in its reviews of the exposure and health literature.

To make the literature reviews accessible to various audiences, HEI Energy, and the Committee developed a video describing the epidemiology literature review and a fact sheet describing the content and potential applications of the epidemiology and exposure literature reviews.

#### 2.3.2 Selecting Research for Funding

Participants agreed that UOGD exposure and health research should consider industry trends and other factors that affect the magnitude and temporal and spatial variability of UOGD chemical releases; distinguish potential UOGD exposures from other sources of exposure including conventional OGD; design studies so that results are broadly applicable across major oil‐ and natural gas‐producing regions of the United States, and, where feasible, use existing data instead of funding collection of new data. They sought research that provides a scientific basis for decision‐making (e.g., setback distances separating UOGD from residences, schools, and other sensitive land uses) and expressed an urgent need for research involving multisector partnerships that can bring about actionable results to protect public health. The stakeholders also noted the importance of including community perception of risk as a valid consideration in identifying research priorities. At the same time, some expressed frustration with continued research and sought immediate action to limit exposures.

#### 2.3.3 Continuing Engagement With Stakeholders

At the workshops, stakeholders made clear that they wanted to stay involved with HEI Energy's research planning and the research itself such as frequent updates on research planning and progress, mechanisms to provide feedback on the research, and practical tools for them to put the research to use.

## 3 HEI Energy's Research Solicitation

The Committee alone is responsible for defining the direction of HEI Energy's research program. It considered all input received at the workshops and findings from its review of the literature to develop HEI Energy's solicitation for research in the form of two Requests for Applications (RFAs).

The Committee noted knowledge gaps about community exposures across oil and gas regions and, from its review of the epidemiology literature, the need for detailed exposure measures. Representatives of all stakeholder groups requested research that not only quantified exposure but identified its specific source so that action could be taken to protect health. For these reasons and others specified in the RFAs, the Committee prioritized funding for research on exposures related to UOGD impacts on air quality, water quality, and noise levels. The Committee apportioned more funding to air quality and noise research because air emissions and noise routinely occur as part of UOGD operations while water releases usually arise under accidental conditions that can be more challenging to study.

The RFA was based on major themes identified in the workshops and literature reviews (Table 1). While the Committee viewed all themes as important, they concluded that research selected for funding must be designed to quantify any links between specific UOGD processes and community exposures, maximize the applicability of results to different regions and conditions, and inform future health studies or risk assessments.

In 2021, HEI Energy awarded $5 million in funding to five research teams who are conducting research to understand the specific UOGD processes that might lead to community exposures through air and water.

## 4 Stakeholder Engagement During Research Implementation

### 4.1 Guiding Principles for Stakeholder Engagement

In response to stakeholder recommendations for effective ongoing engagement, HEI Energy developed “Guiding Principles for Research and Stakeholder Engagement” (“Guiding Principles”) to foster constructive engagement with stakeholders throughout implementation of HEI Energy‐funded research.

Community members may face challenges that take precedence over their participation in workshops and other events. For this reason, the Guiding Principles recommends specific practices to promote equity and access for community members. With the Guiding Principles, investigators are well positioned to:

1. Keep stakeholders abreast of research plans and developments. The Guiding Principles includes a “Stakeholder Engagement Roadmap,” which details a set of practical steps to engage people living in communities where research is occurring, including members of traditionally underrepresented populations and environmental justice communities as defined in the Guiding Principles.
2. Hear and address any stakeholder concerns or questions.
3. Integrate stakeholder and community input into the research plans where appropriate.

### 4.2 Research Team Stakeholder Engagement Plans

Workshop participants expressed a need for research translation and stakeholder involvement throughout this research. Toward this end, the RFA required successful research projects to include stakeholder engagement plans that foster “effective multi‐directional communication with communities living in the areas proposed for study as well as other stakeholders that have an interest in the proposed research.” Successful research teams developed stakeholder engagement plans that feature:

1. Communication strategies to optimize constructive stakeholder engagement and to foster relationships among the research team, community members, industry representatives, government officials, and other local stakeholders;
2. Approaches ensuring that research translation and communication of study designs and results occur through culturally appropriate means;
3. Plans for effective engagement at key intervals during the research program; and
4. Expected outcomes from implementation of the Stakeholder Engagement Plan.

### 4.3 HEI Energy and Research Team Collaboration on Stakeholder Engagement

The Committee recognized the importance of not only ensuring that research is of the highest quality, but also engaging with stakeholders across regions consistently and equitably. For this reason, the Committee recommended that HEI Energy partner with the research teams to confirm that individual research teams follow their proposed plans, coordinate engagement across research teams and study locations to ensure equity and consistency, participate in research team‐led stakeholder engagement events, and work with investigators to ensure that interim and final findings are communicated clearly and consistently for a general audience. Early HEI Energy support has included organizing a peer learning session, hosting initial Open Houses in study locations, and developing a centralized research webpage on its website. In addition, HEI Energy is facilitating engagement by connecting research teams with their own network of stakeholders and providing a centralized mechanism for stakeholders to learn about the funded research.

#### 4.3.1 Peer Learning Session

HEI Energy and the Consensus Building Institute (CBI), a consulting company that specializes in facilitating stakeholder engagement, hosted a peer learning session. The session was facilitated by CBI and provided an opportunity for the research teams to learn from each other based on their variable levels of experience with community‐engaged research. The session helped HEI Energy and the research teams to optimize and coordinate stakeholder engagement plans and identify potential challenges and approaches to address them.

#### 4.3.2 Open Houses

HEI Energy hosted initial in-person and virtual Open Houses in study locations in May and June 2022. The Open Houses provided an opportunity for research teams, community members, and other stakeholders to discuss the research and its objectives, ask questions and express concerns, and to open and encourage lines of communication throughout the research study. In accordance with the Guiding Principles, HEI Energy and its investigators employed several strategies to lower barriers to participation, including translation of educational materials into Spanish (based on knowledge of predominant languages spoken in the study location), an on‐site interpreter, coverage of childcare expenses, a handicap accessible venue, free parking and, where available, public transportation. HEI Energy promoted the Open Houses through local news outlets, radio programs, email blasts, and personalized phone calls and emails. Both in‐person and virtual Open Houses were attended by community members, NGOs with related work, local and regional policymakers and regulators, industry operating in the study area, and academics. The research teams' Principal Investigators continue to engage the local community through in‐person and virtual presentations and meetings.

#### 4.3.3 HEI Energy Website

HEI Energy has developed a centralized website for stakeholders to learn about the research, upcoming and past events, and research progress. It includes fact sheets, live‐streaming, slides from past events, links to future events, and quarterly research updates written for a general audience.

#### 4.3.4 Webinars

HEI Energy hosts educational webinars on emerging topics related to UOGD exposure and health to keep its funded investigators and other stakeholders apprized of research developments, opportunities for research synergy, and methods for successful stakeholder engagement (e.g., managing research fatigue among community participants).

## 5 Conclusions

Funding organizations play a key role in advancing, incentivizing, and providing mechanisms to promote community‐engaged, actionable research. Here we describe HEI Energy's research planning and implementation process centered around multisectoral stakeholder engagement, with the goal of producing research that is likely to be used to inform key decisions. Having followed the process, the Committee could be confident that its research funding recommendations addressed both important knowledge gaps in the literature and the priorities of multisector stakeholders, including communities living in study locations.

The stakeholder workshops fostered open and collegial discussion among people representing all sectors and illuminated the priorities on which they agree. The process helped strengthen existing relationships and forged new ones, allowing for continued engagement during research implementation. The process also led to the production of HEI Energy's “Guiding Principles for Research and Stakeholder Engagement,” and its roadmap for building trust and facilitating constructive uptake of research data and results.

At the same time, HEI Energy and the Committee confronted several challenges. As highlighted in previous literature (Anenberg et al., 2020), the process requires substantial effort from those planning the research and from stakeholders. In the context of community Open Houses in specific study locations, building connections with community members and organizations was more challenging than with government officials, NGOs, and other local stakeholders. Going forward, we will strive to meet these populations where they are, both literally and figuratively, share resources that may be useful to them (e.g., webinar invitations and fact sheets), develop an understanding of their priorities, needs, and time constraints, and take this knowledge into account before asking them to participate in research‐related activities. We will continue to provide opportunities for learning and stakeholder engagement and measure progress to improve the utility of HEI Energy's work. We hope that the process and lessons described here provide building blocks for other funding organizations and investigators to involve stakeholders in research planning and implementation and enhance the utility and acceptance of their research.

## Conflict of Interest

The authors of this manuscript are solely employed by HEI Energy, which is supported with joint funding from the U. S. Environmental Protection Agency, ConocoPhillips, ExxonMobil, Halliburton Energy Services, Inc., and the Hillman Foundation. All research funded by HEI Energy is selected, overseen, and reviewed independently of HEI Energy’s sponsors.

## Supporting information

Supporting Information S1

## Data Availability

Summaries of the workshops described here are available on the HEI Energy website at the following links: Unconventional Oil & Natural Gas—First Public Workshop, June 2014, Pittsburgh, PA (HEI Energy, 2014a). Unconventional Oil & Natural Gas—Second Public Workshop, December 2014, Wheeling, WV (HEI Energy, 2014b). Unconventional Oil & Natural Gas—Third Public Workshop, July 2015, Pittsburgh, PA (HEI Energy, 2015). Scoping Meeting for Human Health Study Critique, January 2018, Boston, MA (HEI Energy, 2018a). HEI Research Planning Workshop #1: Understanding Population‐Level Exposures Related to the Development of Oil and Natural Gas from Unconventional Resources, July 2018, Denver, CO (HEI Energy, 2018b). HEI Research Planning Workshop #2: Understanding Population‐Level Exposures Related to the Development of Oil and Natural Gas from Unconventional Resources, September 2018, Austin, TX (HEI Energy, 2018c).
