# Supplementary material for: Bringing Multisectoral and Multidisciplinary Stakeholders Together to Optimize Environmental Health Research
Source: Geohealth. 2023 Feb 20;7(2):e2022GH000746. doi: 10.1029/2022GH000746 (PMC9941472; doi:10.1029/2022GH000746)
Supplement: Supplementary file 1 — Supporting Information S1 [file GH2-7-e2022GH000746-s001.pdf]

AGU PUBLICATIONS

GeoHealth

Supporting Information for

Bringing Multisectoral and Multidisciplinary Stakeholders Together to Optimize Environmental Health Research

A.S. Rosofsky[1], D.J. Vorhees[1]

[1]Health Effects Institute Energy

Contents of this file

Tables S1 to S3

Additional Supporting Information

Table S1. HEI Energy Stakeholder Workshop Participating Organizations (in alphabetical order).
Table S2. Distribution of Workshop Participants Among Stakeholder Groups
Table S3. HEI Energy Stakeholder Workshop Charge Questions

Introduction

The Supplemental Information contained here summarize additional information about the HEI Energy Stakeholder Workshops.

Table S1. HEI Energy Stakeholder Workshop Participating Organizations (in alphabetical order).

**Academia**	
Boston University	University of California at Berkeley
Carnegie Mellon University	University of Minnesota
Colorado State University	University of Pennsylvania
Drexel University	University of Pittsburgh Graduate School of Public Health
Duquesne University	University of Texas Austin
Emory University	University of Texas Health San Antonio
Georgia Tech	University of Texas Health Science Center at Houston
Juniata College	University of Toronto Dalla Lana School of Public Health
Missouri University of Science and Technology	University of Washington
Ohio State University	Utah State University
South Dakota School of Mines & Technology	Vanderbilt University
South Dakota State University	Virginia Tech
The Pennsylvania State University	Washington & Jefferson College
Texas A&M University	West Virginia University Health Sciences Center
UCLA Institute of the Environment and Sustainability	Wheeling Jesuit University
University of Cincinnati	Wilkes University
University of Colorado	Yale School of Public Health
University Corporation for Atmospheric Research	
**Consulting Companies and Law Firms**	
Adamantine Energy	Parker Environmental and Consulting, LLC
ALL Consulting	Ramboll
Baker Tilly, LLP	Stantec
Billman Geologic Consultants, Inc.	
**Foundations**	
Claude Worthington Benedum Foundation	Richard King Mellon Foundation
Henry L. Hillman Foundation	The Pittsburgh Foundation
**Government - Federal**	
Health Canada	U.S. Department of Agriculture Forest Service
National Institute for Occupational Safety and Health	U.S. Department of Energy National Energy Technology Laboratory
National Oceanic and Atmospheric Administration	U.S. Geological Survey
National Center for Atmospheric Research	U.S. Environmental Protection Agency-Headquarters, Region 8, Region 6
National Institute of Environmental Health Sciences	

Table S1. HEI Energy Stakeholder Workshop Participating Organizations (in alphabetical order).

**Government - State and Local**	
Allegheny County Health Department	Texas Commission on Environmental Quality
Colorado Department of Public Health and Environment	Western States Air Resources Council
Commonwealth of Pennsylvania, Office of Governor Tom Corbett	West Virginia Department of Environmental Protection
Marshall County Emergency Management	Wheeling-Ohio County Health Department
Oklahoma Department of Environmental Quality	
**Nongovernmental Organization**	
American Lung Association	Houston Advanced Research Center
American Petroleum Institute	Investor Environmental Health Network
Breathe Easy Susquehanna County	League of Women Voters Pennsylvania
Carnegie Museum of Natural History	Marcellus Shale Coalition
Center for Epidemiology and Global Health	Moms Clean Air Task Force
Center for Responsible Shale Development	PennFuture
Citizens for Pennsylvania's Future	Pennsylvania Environmental Council
Clean Air Task Force	RAND Corporation
Clean Water Action	Resources for the Future
Earthworks	Southwest Pennsylvania Environmental Health Project
Electric Power Research Institute	Stroud Water Research Center
Endocrine Disruption Exchange	The Nature Conservancy
Environmental Defense Fund	Union of Concerned Scientists
FracTracker Alliance	
**Oil and Natural Gas Industry**	
Anadarko Petroleum Corporation	ExxonMobil American Petroleum Institute
BP	Gulfport Energy Corporation
Chevron	Halliburton
ConocoPhillips	Noble Energy
Consol Energy	Pioneer Natural Resources
EnCana	Shell Oil Company
Equinor	Schlumberger
EQT Corporation	XTO Energy

Table S2. Distribution of Workshop Participants Among Stakeholder Groups

Workshop	Academia	Federal, State, and Local Government	Oil and Gas Industry	Nongovernmental Organizations	Consultants and Legal Firms	HEI Staff	Foundation
Jun-14	38%	16%	10%	22%	3%	10%	2%
Dec-14	29%	14%	14%	20%	6%	9%	7%
Jul-15	31%	12%	20%	19%	7%	8%	3%
Jan-18	22%	9%	29%	25%	4%	11%	0%
Jul-18	27%	18%	28%	15%	2%	9%	1%
Sep-18	29%	18%	33%	8%	4%	8%	0%
min:	22%	9%	10%	8%	2%	8%	0%
max:	38%	18%	33%	25%	7%	11%	7%

Table S3. HEI Energy Stakeholder Workshop Charge Questions

**Charge questions related to understanding human health effects**
1) What information should the committee review to assess the epidemiological literature related to the onshore development of oil and natural gas from unconventional resources?
2) What criteria should the committee use to evaluate study quality?
3) What do you see as key contributions that the Committee can make to the science and the public dialogue around the development of oil and natural gas from shale and other unconventional resources?
4) Looking beyond the initial Human Health Study Critique task, what do you see as key contributions from the Committee's review of literature and research planning in Year 1 and beyond?
**Charge questions related to understanding human exposures**
1) What does the literature tell us?
2) What does the literature not tell us?
3) What are your recommendations for criteria for prioritizing research?
4) What are your recommendations for population-level exposure research?
5) What are your recommendations for further literature review and data analysis before the next workshop to help in defining research priorities?
6) How can existing data be leveraged to understand potential exposures from UOGD?
7) What potential UOGD exposures are not well understood with existing data and merit original research?
8) What exposure pathways and phases of UOGD should be the focus?
9) In conducting such research, what are your recommendations for considering regulatory, environmental, and industry practice variability over time and across regions?
10) What is the value of the research for decision-makers (e.g., regulators, industry, community members, and scientists conducting research)?
